# Berberine Improves Intestinal Motility and Visceral Pain in the Mouse Models Mimicking Diarrhea-Predominant Irritable Bowel Syndrome (IBS-D) Symptoms in an Opioid-Receptor Dependent Manner

**DOI:** 10.1371/journal.pone.0145556

**Published:** 2015-12-23

**Authors:** Chunqiu Chen, Meiling Lu, Qiuhui Pan, Jakub Fichna, Lijun Zheng, Kesheng Wang, Zhen Yu, Yongyu Li, Kun Li, Aihong Song, Zhongchen Liu, Zhenshun Song, Martin Kreis

**Affiliations:** 1 Shanghai Tenth People’s Hospital, Tongji University School of Medicine, Shanghai, China; 2 Department of Biochemistry, Medical University of Lodz, Lodz, Poland; 3 Department of Pathophysiology, Institute of Digestive Disease, Tongji University School of Medicine, Shanghai, China; 4 Charité University Medicine, Department of General-, Visceral- and Vascular Surgery, Campus Benjamin Franklin, Berlin, Germany; University of California, Los Angeles, UNITED STATES

## Abstract

**Background and Aims:**

Berberine and its derivatives display potent analgesic, anti-inflammatory and anticancer activity. Here we aimed at characterizing the mechanism of action of berberine in the gastrointestinal (GI) tract and cortical neurons using animal models and in vitro tests.

**Methods:**

The effect of berberine was characterized in murine models mimicking diarrhea-predominant irritable bowel syndrome (IBS-D) symptoms. Then the opioidantagonists were used to identify the receptors involved. Furthermore, the effect of berberineon opioid receptors expression was established in the mouse intestine and rat fetal cortical neurons.

**Results:**

In mouse models, berberine prolonged GI transit and time to diarrhea in a dose-dependent manner, and significantly reduced visceral pain. In physiological conditions the effects of berberine were mediated by mu- (MOR) and delta- (DOR) opioidreceptors; hypermotility, excessive secretion and nociception were reversed by berberine through MOR and DOR-dependent action. We also found that berberine increased the expression of MOR and DOR in the mouse bowel and rat fetal cortical neurons.

**Conclusion:**

Berberine significantly improved IBS-D symptoms in animal models, possibly through mu- and delta- opioid receptors. Berberine may become a new drug candidate for the successful treatment of IBS-D in clinical conditions.

## Introduction

Berberine is present in several plant species, which are common in the Eastern hemisphere. It has a long history in traditional Chinese medicine, where it has been used to treat diarrhea and gastroenteritis due to its anti-microbial, anti-motility and anti-secretory properties. Berberine and its derivatives also display a potent analgesic[1], anti-inflammatory[2] and anticancer activity [3];they also have a potential therapeutic effect on diabetes [4], hyperlipidemia[5], cardiovascular diseases [6], and central nervous system (CNS) disorders[7]. Of note, the antidepressant-like activity of berberine involves inhibition of monoamine oxidase [8].However, sigma-1 receptor and nitric oxide signaling may also be targeted by berberine[9]. Recently, progression of visceral hypersensitivity to colorectal distension controlled by berberine has been reported[1]. The antinociceptive and antidepressant-like activity of berberine underlines its therapeutic potential for the treatment of patients with irritable bowel syndrome (IBS)[10,11].Although we found that [12] berberine plays an inhibiting role on gastrointestinal motility in rodents, which is closely related to that of the endogenous opioid system, the mechanism has not been fully clarified by now.

Mu opioid receptors (MORs), which belong to the opioid receptor family, are involved in diverse physiological and pathological states, and MOR agonists were found to produce analgesia, alleviate depression symptoms, and relieve stress and anxiety [13].Furthermore, genetic deletion of MOR and delta opioid receptor (DOR), but not kappa opioid receptor (KOR)as shown to increase anxiety symptoms and depressive-like behavior [14]. On the other hand, opioid receptor agonists were found to attenuate visceral hyperalgesia and inhibit an enhanced excitability of colonic sensory neurons [15]. MOR activation reduces persistent pain and improves negative emotional states. Moreover, clinical trials have been initiated to assess the effectiveness of MOR agonists in chronic pain and depression[16].

DOR has high sequence similarity to MOR, yet different physiological and pharmacological property. As reviewed by Pradhan et al.[17], preclinical data confirmed that DOR activation reduces persistent pain and improves negative emotional states.

The aim of this study was to assess the efficacy of berberine in relieving IBS-D symptoms in mice through employing currently available animal models of hypermotility, excessive secretion and abdominal pain. We also explored the mechanism of action of berberine in the mouse GI tract and characterized opioid receptor expression after berberine treatment in mouse bowel and rat fetal cortical neurons. We conclude by suggesting that berberine produces potent antinociception and reverses GI hypermotility in an opioid-receptor dependent manner.

## Methods

### Animals

Male BALB/c mice, weighing 25-30g and female Sprague-Dawley (SD) rats pregnant for 18 days were purchased from the Experimental Animal Center of Tongji University, Shanghai, China. Animals were housed at a constant temperature of 22°C and kept at a constant photoperiod (12:12-h light-dark cycle) in sawdust-lined plastic cages with free access to standard laboratory irradiated chow (purchased from XieTong company, China)and tap water. All operations were performed under aseptic conditions. Experimental procedures complied with international guidelines for care and use of laboratory animals and were approved by the Animal Ethics Committee of Tongji University, Shanghai, China.

### Whole gut transit time

In the morning (7 a.m.) of each experimental round, mice were transferred to individual empty plastic cages (devoid of bedding) and were left to acclimatize to the cage for 1 h. Mice were then injected *i*.*p*. with vehicle (saline) or berberine (0.5, 1, 2 or 5mg/kg) and 15 min later gavaged with 150 ml of prewarmed (37°C) Evans blue marker (5% Evans blue, 5% gum Arabic in drinking water). Mice were returned to their individual cages. The time from the end of gavaging to the first blue faecal pellet was measured in minutes and constituted the whole gut transit time. In further experiments the non-selective opioid receptor antagonist naloxone (NAL, 1mg/kg), and selective MOR(β-funaltrexamine, β-FNA,1mg/kg),DOR (naltrindole, NTI, 1 mg/kg) or KOR (nor-binaltorphimine, nor-BNI,10mg/kg) antagonists were administered *i*.*p*. 15 min prior to vehicle or berberine (BER, 2mg/kg).

### Castor oil-induced diarrhea

Berberine (0.5 or 1 mg/kg) or vehicle (saline) were injected *i*.*p*. 15 min before *p*.*o*. administration of castor oil (0.2 ml/mouse) and the time elapsed until unformed watery stools appeared was recorded.

The non-selective opioid receptor antagonist naloxone (1 mg/kg)and the selective opioid receptor antagonists were administered *i*.*p*. 15 min prior to berberine (0.5 mg/kg, *i*.*p*.) treatment.

### Stress-induced hypermotility

One day before the experiment, mice were divided into two groups: control and stressed by the novel environment (NE-stressed). Animals from the control group were separated from each other and placed into individual cages with free access to chow and water to accommodate overnight. On the day of the experiment, mice received berberine (0.5 and 1.0 mg/kg, *i*.*p*.) or vehicle. Fifteen minutes later all animals were placed in individual cages without access to food and water. One hour later, the number of faecal pellets in each cage was counted.

### Mustard oil and capsaicin-induced visceral pain

Behavioral pain-related responses to intracolonic (*i*.*c*.) administration of mustard oil (MO) and capsaicin solution were determined as described previously[18]. Fifteen minutes after *i*.*p*. injection of berberine (0.5 and 1 mg/kg in MO test and 0.5, 1, and 5mg/kg in capsaicin test), 50 μL of MO (1% vol/vol dissolved in 70% ethanol) or capsaicin (0.3% w/v in 10% ethanol-10% Tween 80–80% saline) were administered into the colon of anesthetized mice using a fine catheter (external diameter 0.61 mm, 4 cm long, Minipack, Portex, Hythe, UK). Vaseline was applied to the perianal area to avoid the stimulation of somatic areas by contact with MO or capsaicin. After the administration of MO or capsaicin, the animals were placed in individual plastic cages in a quiet environment. Five minutes later, spontaneous pain-related responses: licking of the abdomen, stretching the abdomen, squashing the lower abdomen against the floor, and abdominal retractions were counted for 20 min.

The non-selective opioid receptor antagonist naloxone (1 mg/kg) and the selective opioid receptor antagonists were administered *i*.*p*. 15 min prior to berberine in the MO induced pain test.

### Behavioral responses to acetic acid induced pain test

The acetic acid test was performed in the morning as described recently[18]. Fifteen minutes after *i*.*p*. injection of berberine (0.5, 1 and 5 mg/kg), mice received an *i*.*p*. injection of an acetic acid solution (0.5%, vol/vol in 0.9% NaCl). Animals were then placed individually in empty cages and after 5 min abdominal stretchings were counted for 15 min in 5 min intervals. A typical stretch was characterized by an elongation of the body and the development of tension in the abdominal muscles and hind paws. Vehicle (saline) was used in control experiments.

### Expression of opioid receptors

#### Study material

To investigate the effect of berberine on opioid receptor expression in the ileum and the colon, mice were injected with berberine (0.5, 1, 2 and 5mg/kg,*i*.*p*.) and 45 min later sacrificed by cervical dislocation. Full-thickness segments of ileum and colon were removed and divided into two groups; one was kept in -80°C for genetic analysis and the other saved in 4% PFA for histological evaluation.

The expression of opioid receptors at mRNA level was also evaluated in fetal cortical neurons. Briefly, whole Sprague Dawley rat fetus brains were removed and cortices dissected in HBSS and trypsinized (0.25% trypsin in PBS at 37°C for 12 min)[19].Trypsin was then neutralized by DMEM/F12 with 10% FBS. Dissociated cells were filtered through 40 μm meshes (BD Biosciences, Bedford, MA) and plated in 35or 60mm dishes pre-treated for at least 2 h with poly-D-Lysine (Sigma, St. Louis, MO). After incubation for 18-24h, the non-adherent cell suspension was discarded and neurobasal media supplemented with 2% B27 (Invitrogen, Carlsbad, CA, USA) was added. Neurons were cultured for 7 days to collection. Berberine(10^-8^mol/L) was added and cells maintained in culture for the 0.5, 1, and 2 days prior to material collection.

#### RNA extraction and real-time RT-PCR

Total RNA was isolated from fetal rat primary neurons using TRIzol solution (Invitrogen, Carlsbad, CA, USA) in accordance with the manufacturer's instructions. Reverse transcription was performed in a 10 μl reaction system with 1 μg total RNA treated with RT-PCR kit to synthesize first-strand cDNA (TAKARA, Japan). The resulting cDNA was amplified using KAPA qPCR Master Mix (KAPA, Boston, USA) and the following primers(Invitrogen, USA):

OPRM (for mouse or rat MOR): 5'- CAACTTGTCCCACGTTGATG -3' (F)

and 5'- TCCAAAGAGGCCCACTACAC -3' (R);

OPRD (for mouse or rat DOR): 5'- TTGGCTTCAGGTGTTGGGGT -3' (F)

and 5'- GCGAAGAGGAACACGCAGAT -3' (R);

OPRK (for mouse or rat KOR): 5'- GCAGCCTGAATCCTGTTCTC -3' (F)

and 5'- TCATCCCTCCCACATCTCTC -3' (R);

GAPDH (housekeeping gene): 5'-GGTGAAGGTCGGTGTGAACG-3' (F)

and 5'- GTGAAGACACCAGTAGACTC -3' (R).

### Drugs

Naloxone, naltrindole, β-funaltrexamine, nor-binaltorphimine and capsaicin were obtained from Tocris Bioscience (Tocris, Ellisville, Missouri, USA). Allyl isothiocyanate (mustard oil, MO) was purchased from Merck (Darmstadt, Germany). Berberine was purchased from Shanghai Shifeng Biotechnology Ltd., China. All drugs were dissolved in dimethyl sulfoxide (DMSO) and diluted in 0.9% saline to final concentrations.

### Statistics

PRISM 5.0 (GraphPad Software Inc., La Jolla, CA, USA) was used for statistical analyses. One-way analysis of variance (ANOVA) followed by Student-Newman-Keuls *post hoc* test was used for analysis of multiple treatment means. *P* values < 0.05 were considered significant. The data are expressed as means ± SE.

## Results

### Berberine inhibits gastrointestinal transit in mice in an opioid receptor-dependent manner

**Fig 1:**
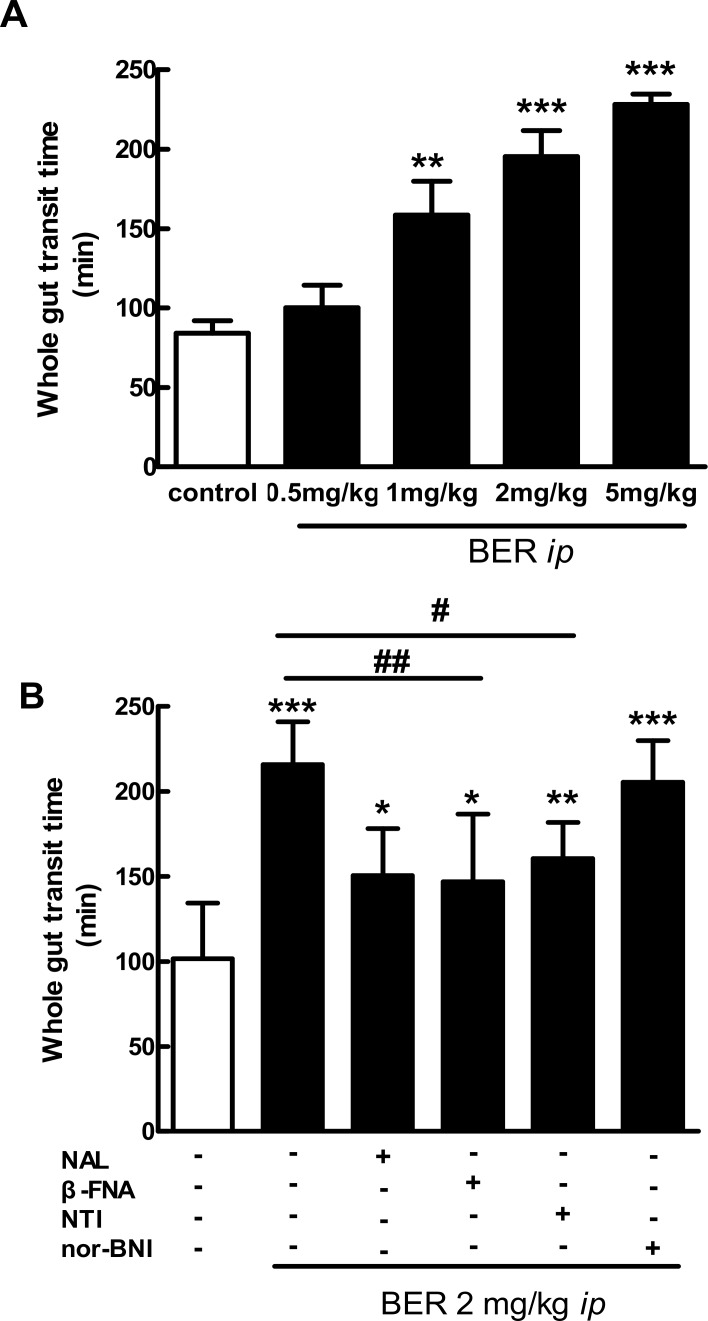
Effect of berberine (BER) alone or in presence of opioid antagonists on whole gut transit time in mice. Data are mean ± SE (*n* = 6–10). A: *I*.*p*. berberine (0.5, 1, 2 and 5 mg/kg) prolonged the whole gut transit time in a dose-dependent manner. **P < 0.01, ***P < 0.001 *vs*. control. B: *I*.*p*. naloxone (NAL, 1 mg/kg), β-funaltrexamine (β-FNA,1 mg/kg) and naltrindole (NTI, 1 mg/kg), but not nor-binaltorphimine (nor-BNI,10 mg/kg) blocked the effects of berberine(BER, 2 mg/kg, *i*.*p*.) on the whole gut transit time. ^*^ *P* < 0.05, ^**^ *P* < 0.01 and ^***^ *P* < 0.001, *vs*. control; ^#^ *P* < 0.05 and ^##^ *P* < 0.01, berberine *vs*. berberine + antagonists.

As shown in Fig 1A, berberine(0.5, 1, 2 and 5 mg/kg, *i*.*p*.) prolonged the whole gut transit time in mice in a dose-dependent manner. The effect of berberine(2 mg/kg, *i*.*p*.) was blocked by the *i*.*p*. administration of the non-selective opioid receptor antagonist naloxone, the selective MOR antagonist β-funaltrexamine and DOR antagonist naltrindole, but not KOR antagonist nor-binaltorphimine (Fig 1B).

### Berberine reduces diarrhea and gastrointestinal hypermotility in mice

**Fig 2:**
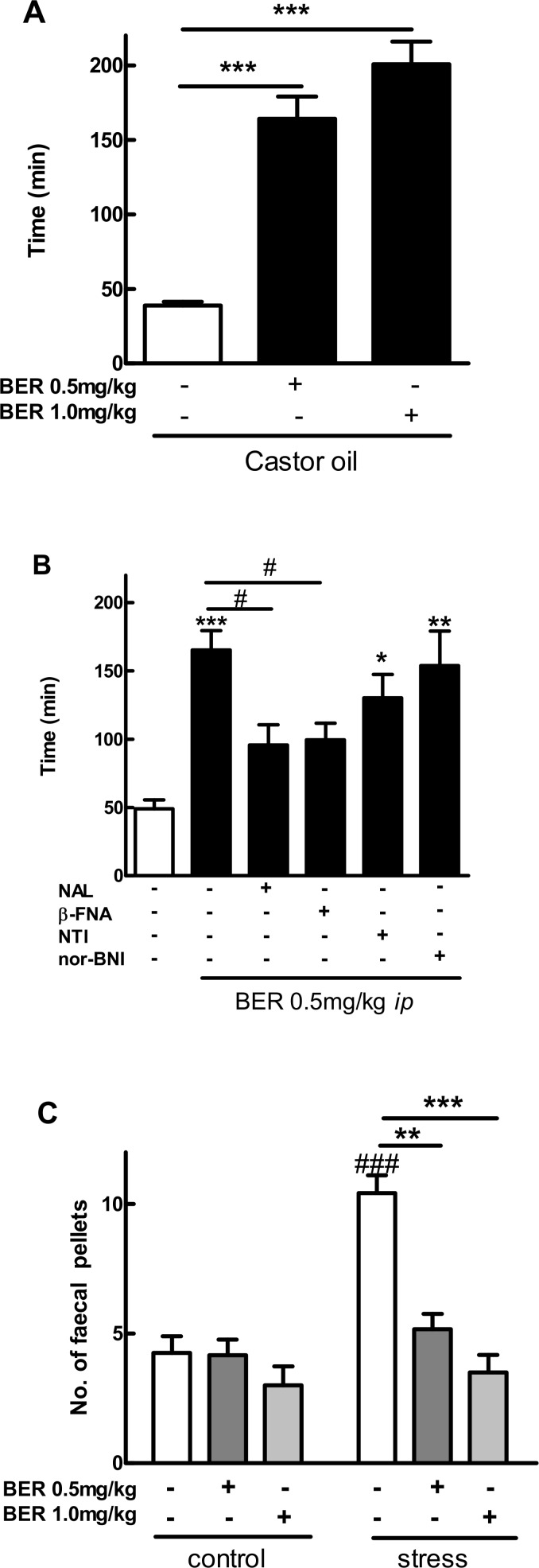
Effect of berberine (BER) on diarrhea and gastrointestinal hypermotility in mice. Data are mean ± SE (*n* = 6–10). A: *I*.*p*. berberine (0.5 and 1 mg/kg) prolonged time to diarrhea in the mouse model induced by oral administration of castor oil. ***P < 0.001, *vs*. control. B: *I*.*p*. opioid antagonists: naloxone (NAL, 1 mg/kg) and β-funaltrexamine (β-FNA,1 mg/kg), but not naltrindole (NTI, 1 mg/kg) or nor-binaltorphimine (nor-BNI,10 mg/kg) blocked the effects of berberine(BER, 0.5 mg/kg, *i*.*p*.) in the mouse model of castor oil-induced diarrhea. *P < 0.05, **P < 0.01, ***P < 0.001, *vs*. control; ^#^ *P* < 0.05, berberine + antagonists *vs*. berberine alone. C: *I*.*p*.berberine (0.5 and 1 mg/kg)reduced the number of fecal pellets in the mouse model of hypermotility induced by NE (novel environment)-related stress. ^###^ *P* < 0.001, non-treated NE-stressed *vs*. non-treated control; **P < 0.01, *** *P* < 0.001, *vs*. non-treated NE-stressed.

Berberine(0.5 and 1 mg/kg,*i*.*p*.) significantly prolonged time to diarrhea in a mouse model induced by oral administration of castor oil (Fig 2A). The effect of berberine(0.5 mg/kg, *i*.*p*.) was blocked by the *i*.*p*. administration of naloxone and the selective MOR antagonist β-funaltrexamine, but not DOR or KOR antagonists (Fig 2B).

At last as shown in Fig 2C, berberine(0.5 and 1 mg/kg, *i*.*p*.) significantly reduced the number of fecal pellets in the mouse model of GI hypermotility induced by novel environment (NE)-related stress.

### Antinociceptive effect of berberine in mouse models of pain

**Fig 3:**
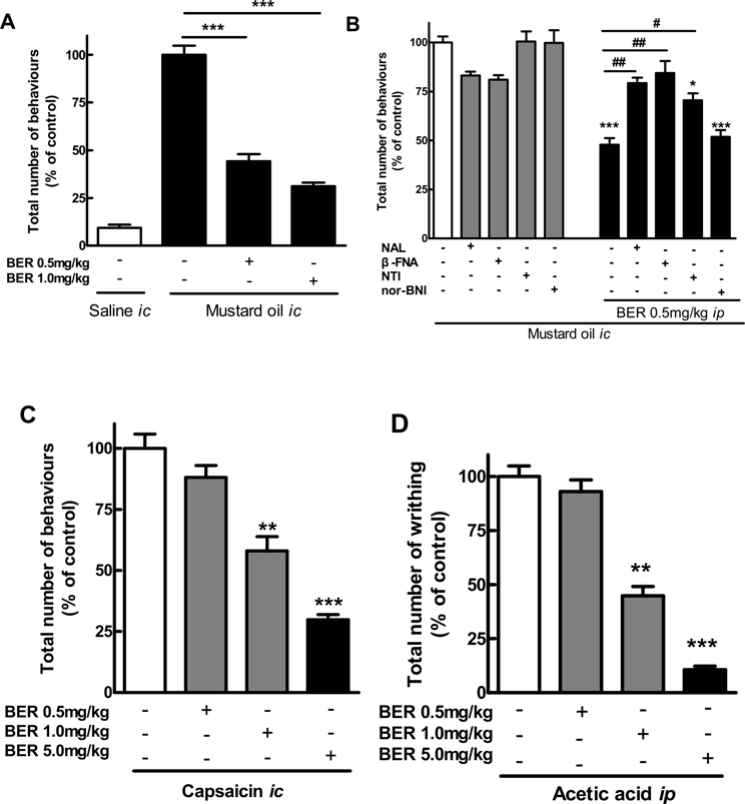
Antinociceptive effect of berberine (BER) in mouse models of pain. Data are mean ± SE (*n* = 6–10).A: *I*.*p*. berberine (0.5 and 1 mg/kg)reduced the number of pain-related behaviors in the mustard oil (*i*.*c*.)-induced model of visceral pain. ***P < 0.001, *vs*. control. B: *I*.*p*. opioid antagonists naloxone (NAL, 1 mg/kg), β-funaltrexamine (β-FNA,1 mg/kg) and naltrindole (NTI, 1 mg/kg), but not nor-binaltorphimine (nor-BNI,10 mg/kg)blocked the effects of berberine(BER, 0.5 mg/kg, *i*.*p*.)on the number of pain-related behaviors in the mustard oil model.*P < 0.05; ***P < 0.001, *vs*. control; ^#^ *P* < 0.05; ^##^ *P* < 0.01, berberine + antagonists *vs*. berberine alone. C: *I*.*p*. berberine (0.5, 1 and 5 mg/kg)reduced the number of pain-related behaviors in the capsaicin-induced model of visceral pain. **P < 0.01; ***P < 0.001, *vs*. control. D: *I*.*p*. berberine (0.5, 1 and 5 mg/kg)reduced the number of pain-related writhing induced by *i*.*p*. administration of acetic acid.**P < 0.01; ***P < 0.001, *vs*. control.

Berberine(0.5 and 1 mg/kg, *i*.*p*.) significantly reduced the number of pain-related behaviors in the mouse model of MO-induced visceral pain (Fig 3A).Interestingly, the effect of berberine(0.5 mg/kg, *i*.*p*.) was blocked by the *i*.*p*. administration of the non-selective opioid receptor antagonist naloxone and the selective MOR and DOR antagonists (β-funaltrexamine and naltrindole, respectively) (Fig 3B).

Moreover, the *i*.*p*. administration of berberine(0.5, 1 and 5 mg/kg)produced a potent, dose-dependent antinociceptive effect in the capsaicin (*i*.*c*.) and acetic acid (*i*.*p*.) (Fig 3C and 3D, respectively) mouse model of visceral pain.

### Berberine changes opioid receptor expression in mouse intestine

**Fig 4:**
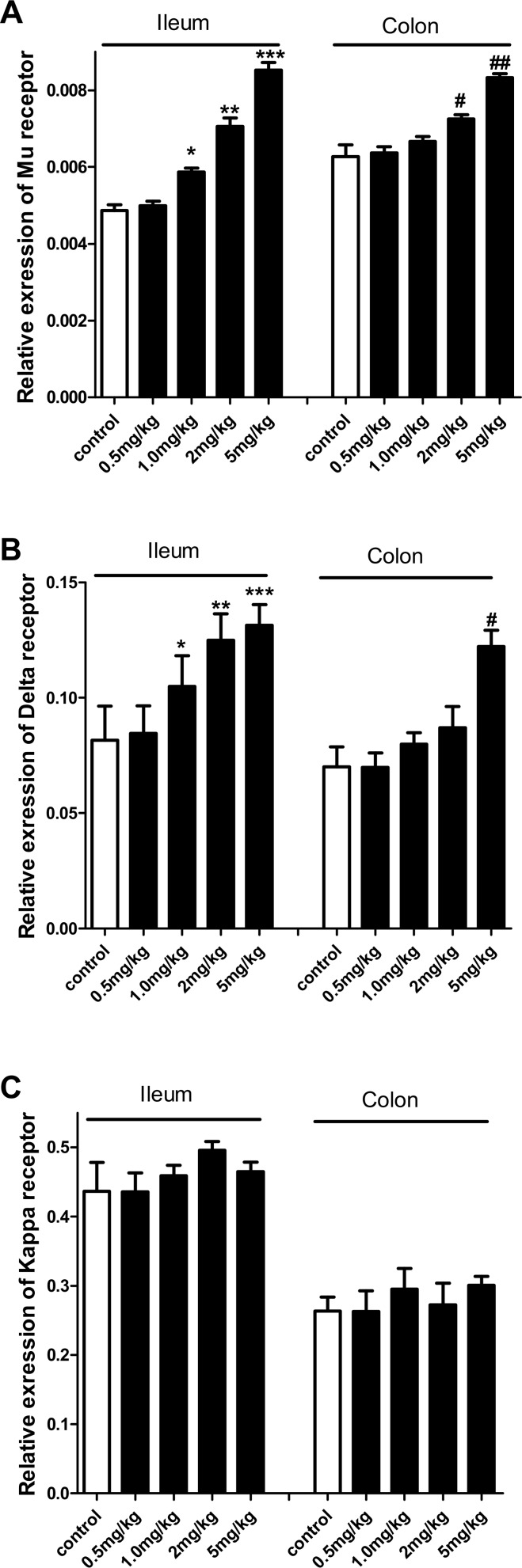
Effects of berberine (BER) on opioid receptor expression in the mouse ileum and colon. A: Berberine *i*.*p*. (1, 2 and 5 mg/kg) increased MOR mRNA expression in the mouse ileum in a dose-dependent manner, and berberine *i*.*p*. (2 and 5 mg/kg) increased MOR expression in the mouse colon. *P<0.05,**P<0.01 and ***P<0.001, *vs*. ileum control; ^#^ *P*<0.05, ^###^ *P*<0.001, *vs*. colon control. B: Berberine *i*.*p*. (1, 2 and 5 mg/kg) increased DOR mRNA expression in the mouse ileum in a dose-dependent manner, and berberine *i*.*p*. (5 mg/kg) increased MOR expression in the mouse colon. *P<0.05, **P<0.01 and ***P<0.001, *vs*. ileum control; ^#^ *P*<0.05 *vs*. colon control. C: Berberine *i*.*p*. (0.5, 1, 2 and 5 mg/kg) had no effect on KOR mRNA expression in the mouse ileum and colon.

Berberine at the dose of 1, 2 and 5mg/kg increased MOR mRNA expression in the mouse ileum and at the dose of 2 and 5 mg/kg in the colon (Fig 4A). DOR expression was also increased in the mouse GI tissues; however, in the colon only the highest dose of berberine (5 mg/kg) produced statistically significant results (Fig 4B). No effect of berberine on KOR mRNA expression was found in the mouse GI tract (Fig 4C).

### Berberine changes opioid receptor expression in rat brain cortex

Previous studies have demonstrated that opioid receptor agonists affect neuronal activity in the CNS [20]; furthermore, primary culture of cortical neurons is widely used in the research on neurological disorders[21].However, owing to difficulties in dissection and culture of mouse fetal cortical neurons, a universally accepted protocol for their derivation has not yet been determined. As a result, the effect of berberine was examined on cultured rat fetal cortical neurons.

**Fig 5:**
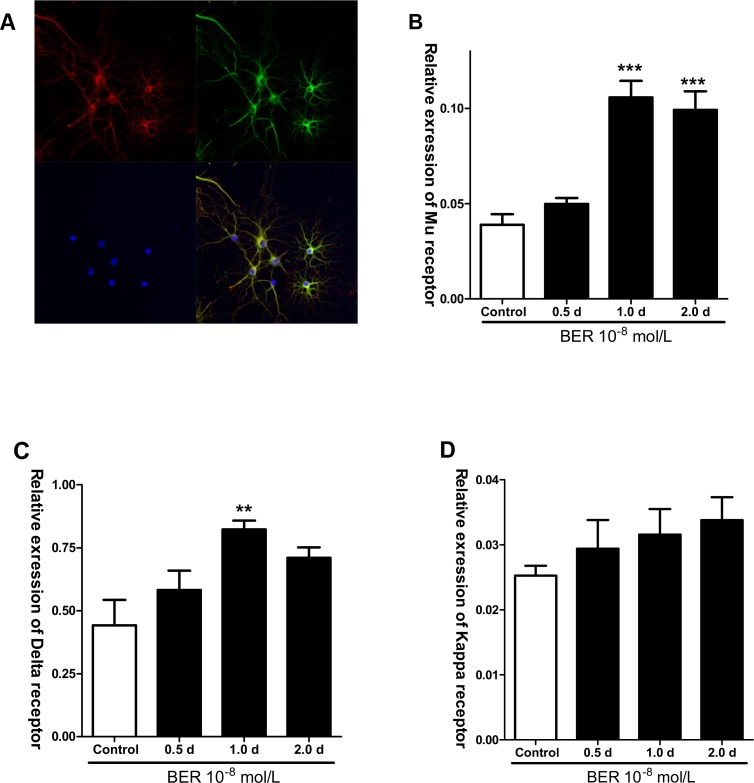
Effects of berberine (BER) on opioid receptors expression of mRNA level in rat fetal cortical neurons. A: Fetal cortical cells cultured for 7 days expressed MOR (β-tubulin^+^ in red, MOR^+^ in green, DAPI^+^ in blue and merged). B: Berberine (BER, 10^-8^mol/L) significantly increased MOR mRNA expression when added 1.0 and 2.0 day before cell collection in 7-day culture (***P<0.001 compared with control). C: Berberine (BER 10^-8^mol/L) increased DOR mRNA expression when added1.0day before cell collection in 7-day culture. *P<0.05 compared with control. D: Berberine had no effect on KOR expression in 7 day cortical neuron cultures.

As shown in Fig 5A, nearly all rat fetal cortical neurons were β-tubulin, MOR and DAPI-positive after7 days of culture. Determination of neuronal purity using dark field imaging showed that the percentage of β-tubulin-immunostained neurons was over 95%. Incubation of cortical neurons with berberine (10^−8^ mol/L, added to a 7-day culture 1 and 2 days before collection) significantly increased the mRNA expression of MOR compared with the control group (Fig 5B).

Of note, the increase of DOR mRNA expression after berberine(10^-8^mol/L) (only 1 day before collection) treatment was observed in the cortical neurons cultured for 7-day, as compared with control (Fig 5C). No effect of berberine on the mRNA expression of KOR was found in the cortical neuron culture (Fig 5D).

## Discussion

Patients with IBS-D suffer markedly from loose and frequent stools, and from the associated urgency and fear of incontinence, which generate panic and anxiety. Furthermore, mental disorders constitute an important factor in intestinal dysfunction, and the disease itself is often closely related to mental ailments, such as anxiety, anger, depression, and fear [22]. Another main symptom of IBS is abdominal pain, presented in most patients [23].

In this study, we explored the therapeutic potential of berberine in treating IBS-D using mouse models mimicking physiological conditions and disease symptoms. We found that berberine prolonged the whole gut transit time; of note, a classic non-selective opioid antagonist naloxone, as well as selective MOR (β-funaltrexamine) and DOR (naltrindole) blocked the anti-motility effect, suggesting that both receptor types may be involved in regulation of whole gut motility by berberine. This is in line with earlier reports showing that berberine inhibits myoelectrical activity and GI transit through the endogenous opioid system[12].In the recent years, MOR-DOR interactions were studied in detail and it was found that MOR and DOR may form heteromers, if co-expressed[24]. The co-expression of MOR and DOR and functional interaction has been widely reported. For example, MOR was shown to co-internalize with DOR and target lysosomal degradation after treatment with a DOR agonist[25]. Moreover, DOR can alter the functions and the trafficking of MOR[26]. In the present study we showed that the activation of MOR by berberine may simultaneously affect DOR function in physiological conditions. However, berberine prolonged the time to diarrhea in the mouse model induced by orally administered castor oil exclusively in a β-funaltrexamine-dependent manner. These results suggest that MOR is principally responsible for berberine action in pathophysiological conditions.

Earlier reports demonstrate that the activation of MOR and DOR controls pain signaling[27,28]. For example, it was found that peripheral MOR and DOR play a major role in the tonic inhibition of mechanical allodynia[29,30]and MOR and DOR agonists alleviate mechanical pain and relieve heat and mechanical hyperalgesia[31,32]; opioid receptor agonists of different background and chemical structure also produce antinociception in inflammatory or chemically‐induced pain (for example [18,33,34]).In line, our results showed that berberine significantly relieved visceral pain induced by mustard oil, capsaicin and acetic acid which blocked by MOR and DOR antagonists in mustard oil test, confirming crucial role of peripheral MOR and DOR in berberine-mediated action.

By now there have been no studies investigating in detail the interaction of berberine in the endogenous opioid system. Here we showed that the administration of berberine in mice significantly enhanced MOR and DOR mRNA expression in ileum and colon.

Since berberine was shown to easily cross the blood brain barrier after systemic administration[1]and may thus affect CNS, we also investigated the effect of berberine on opioid receptor expression in fetal cortical neurons. Interestingly, MOR expression was significantly enhanced after berberine treatment in 7 days cortical neurons, while DOR was up-regulated also in 7 day neurons.

Earlier reports show that the activation of MOR and DOR reduces depression, possibly as a result of stress or anxiety relief [35,36]. Several studies revealed that already a single treatment with MOR and DOR agonists produce antidepressant-like effects in the forced swimming test, which is one of the most popular animal models for screening antidepressants [37,38]. Furthermore, acute administration of the selective MOR agonists, such as methadone, fentanyl, morphine, hydrocodone, buprenorphine, and nalbuphine in an intracranial self-stimulation (ICSS)blocked both stimulation of stretching and depression produced by lactic acid[39].Recently a novel DOR agonist, KNT-127 demonstrated antidepressant-like and antinociceptive effects in mice without producing convulsions[40]. The fact that berberine simultaneously acts through MOR and DOR, but also enhances their expression in the CNS may have vital implications in the treatment of anxiety symptoms and depressive-like behavior, which are typical for IBS patients. This action must not be overlooked in further, clinical trials of berberine.

Finally, patients with diarrhea-predominant irritable bowel syndrome (IBS-D) suffer markedly from loose and frequent stools, with the associated urgency and a fear of incontinence. Another main symptom of IBS is abdominal pain, presented in most patients. Furthermore, this disease itself is often closely related to mental ailments. According to our berberine mechanism research here, it encourages to use in the anti-IBS treatment. However, further clinical studies are needed to investigate this mechanism and effects in detail.

## Conclusion

Berberine alleviates disease symptoms in mouse models mimicking the symptoms of IBS-D. Further experiments in animal models and in vitro cell cultures suggested that the effect of berberine on bowel motility and visceral pain may be mediated in MOR and DOR-dependent manner. Our data validate further use of berberine as an attractive drug in the treatment of IBS-D, targeting GI symptoms and CNS-related consequences of the disease; we also recommend further clinical studies of this compound.
